# Ascending Thoracic Aortic Dissection: A Case Report of Rapid Detection Via Emergency Echocardiography with Suprasternal Notch Views

**DOI:** 10.21980/J8WW6W

**Published:** 2020-04-15

**Authors:** Brandon Backlund, Anastasia Kendrick-Adey, Rachel Harper, Martin Makela

**Affiliations:** * University of Washington, Department of Emergency Medicine, Seattle, WA

## Abstract

Aortic dissection is a life-threatening, time-sensitive emergency. Conventional diagnostic imaging modalities such as computed tomography (CT) can be time-consuming to obtain, and require that the patient leave the emergency department (ED); as such, they are unsuitable for unstable patients. Emergency focused transthoracic echocardiography (ETTE) is commonly performed in the ED as part of the evaluation of a patient presenting with chest pain, but the suprasternal notch view (SSNV) is much less well-known and infrequently included in this assessment.

We present a case of a 51-year-old previously healthy man who presented to the ED complaining of chest pain that had resolved prior to arrival, and a mild headache. His vital signs were notable for hypotension, but physical exam was unremarkable. Chest x-ray revealed a borderline widened mediastinum. A standard ETTE was within normal limits, but additional SSNV demonstrated a dissection flap in the aortic arch. The patient was taken to the operating room for surgical repair 75 minutes after the ED ultrasound was performed; the operation was successful, and the patient was discharged to home post-operatively with good outcome.

Standard ETTE has limited ability to visualize the ascending aorta and aortic arch. Addition of SSNV allows visualization of these structures and may improve diagnostic accuracy and time to diagnosis of proximal aortic dissection.

**Topics:**

Aortic dissection, emergency echocardiography, point-of-care ultrasound, POCUS, emergency ultrasound, suprasternal notch view.

[Fig f1-jetem-5-2-v14]

**Figure f1-jetem-5-2-v14:**
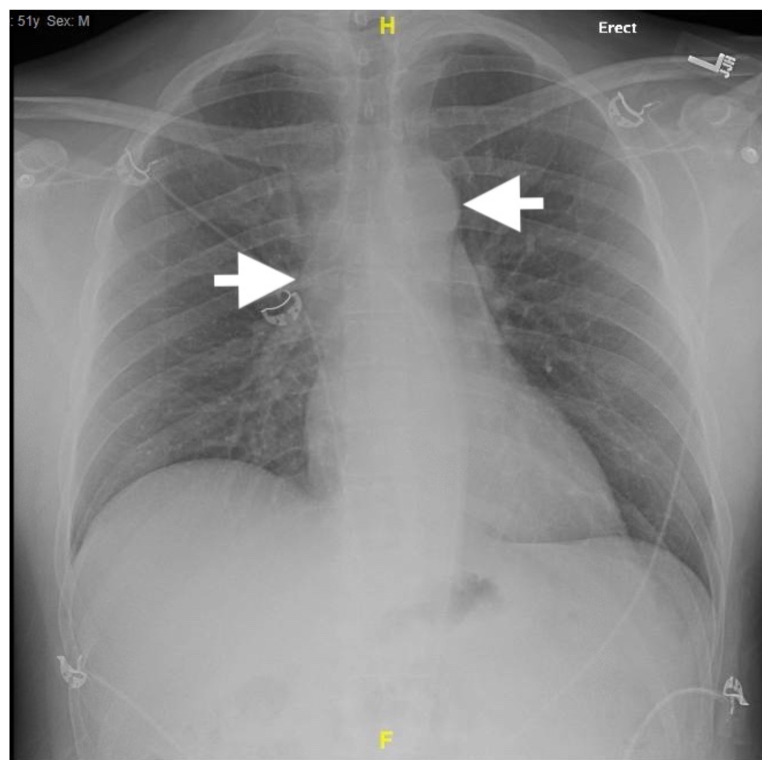

[Fig f2-jetem-5-2-v14]

**Figure f2-jetem-5-2-v14:**
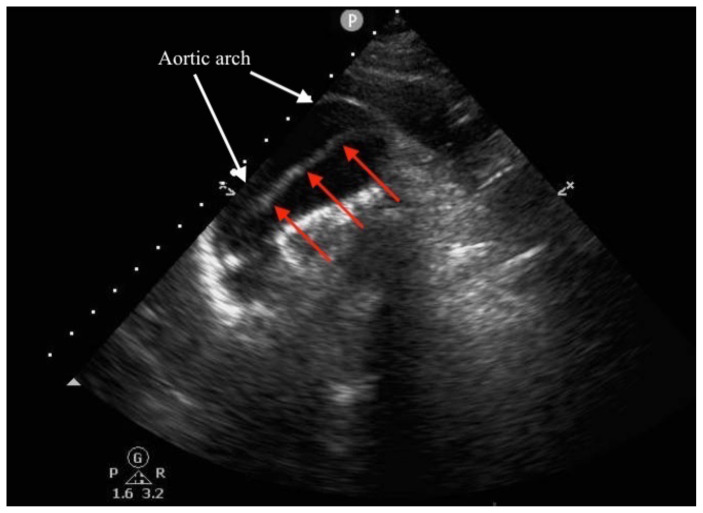

[Fig f3-jetem-5-2-v14]

**Figure f3-jetem-5-2-v14:**
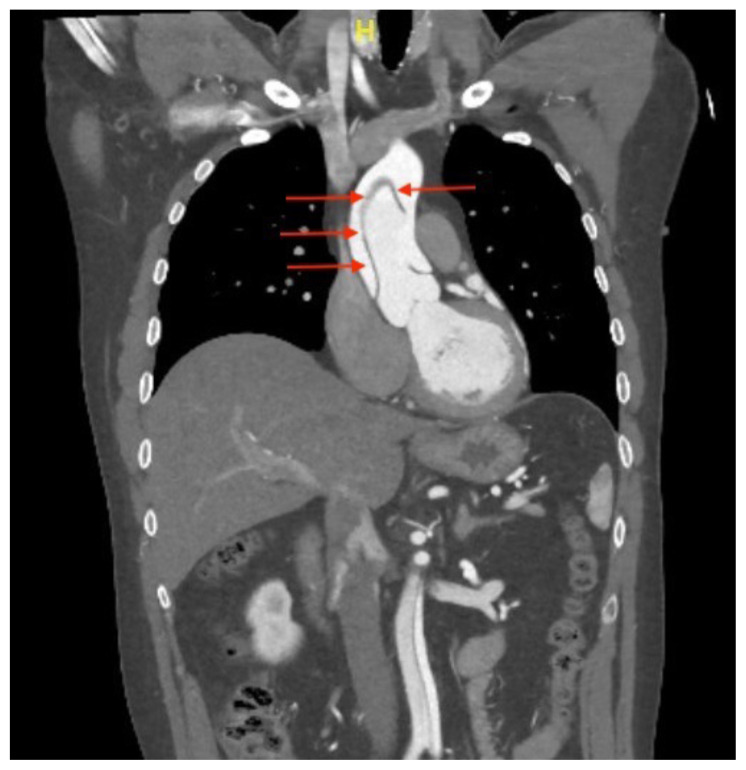

[Fig f4-jetem-5-2-v14]

**Figure f4-jetem-5-2-v14:**
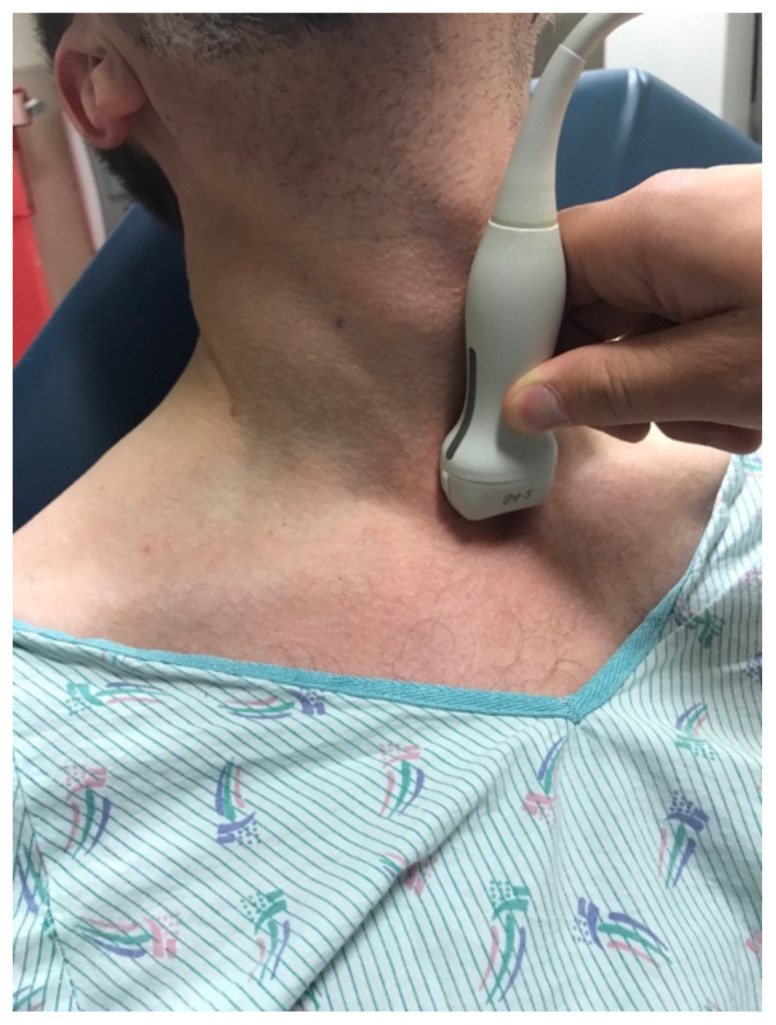
Parasternal Long Video: https://youtu.be/Nfbhj8oEaE0Suprasternal Notch View Video: https://youtu.be/fdqjgsWrxjM

## Introduction

Aortic dissection (AD) is a life-threatening emergency condition with widely variable presentations.^1^,^2^ Time to diagnosis is of the essence because survival decreases proportionately with increasing time to definitive care.^3^ The majority of these patients will present via the Emergency Department (ED), and thus emergency physicians will frequently be the first providers to treat these patients. Clinician-performed focused emergency transthoracic echocardiography (ETTE) is readily available in many EDs. It can quickly provide valuable information about cardiac function in the evaluation of patients presenting with chest pain or other complaints of possible cardiac origin, but standard views offer limited visualization of the proximal aorta. Though not widely utilized, the suprasternal notch view (SSNV) is an additional window that can be performed during ETTE that allows partial or complete visualization of the ascending aorta, aortic arch, and proximal descending aorta, and may facilitate early identification of proximal AD.

## Presenting concerns, clinical findings, patient course

A 51-year-old man was brought to the emergency department (ED) by paramedics for chest pain, headache, and hypotension. He endorsed no significant or chronic health history, reported no prior surgeries, and denied taking any medications routinely. Of note, he had not seen a doctor in many years. The patient reported his chest pain had resolved by the time of ED arrival, but noted a mild headache. On arrival the patient had a normal mentation, was afebrile, with a blood pressure of 85/59 mmHg, a pulse of 59 beats per minute, a normal respiratory rate, and an oxygen saturation of 100% on room air. The physical examination was unremarkable, with clear lung fields bilaterally and normal heart sounds. Electrocardiography was normal. Chest x-ray was notable for a borderline widened mediastinum.

Over his ED course, the patient’s blood pressure improved slightly, but remained borderline hypotensive, with systolic blood pressures ranging between 90–100, despite infusion of a 1,000 mL bolus of crystalloid. However, as the patient remained alert and comfortable, vasopressors were not utilized since his blood pressure did not drop further.

A standard, emergency point-of-care transthoracic echocardiography (ETTE) performed by an emergency medicine resident was notable for grossly normal left ventricular function, no pericardial effusion, and normal-appearing aortic root. However, due to limited visualization of the proximal aorta, the patient’s hypotension, and the widened mediastinum seen on chest x-ray, an additional suprasternal view of the aorta was performed which demonstrated a dissection flap in the ascending aorta and aortic arch.

Cardiothoracic surgery was consulted emergently, and after reviewing the ETTE and the findings on SSNV, the operating room was mobilized while the patient was taken for computed tomography (CT) with contrast of the aorta. This confirmed a type A AD extending from the ascending aorta to the left common iliac artery, with extension into the bilateral carotid arteries. The patient was taken directly to the operating room from the CT scanner, 75 minutes after the ED ultrasound. He underwent successful grafting of the proximal aorta, recovered well post-operatively, and was discharged to home on postoperative day 6 with normal neurologic and cardiac function. He continued to do well at 1-year follow up.

## Significant findings

Chest x-ray demonstrates a widened mediastinal contour (between white arrows). The white arrow on the left marks the outline of the ascending aorta, which can be seen to curve or bulge out as it courses upwards, suggesting dilation or enlargement.

Video of parasternal long-axis bedside transthoracic echocardiogram: The initial images showed grossly normal left ventricular function, and no pericardial effusion or evidence of cardiac tamponade. However, the proximal aorta beyond the aortic valve was poorly-visualized in this window.

Suprasternal notch view of aortic arch: The aortic arch is denoted by the white arrows, and a dissection flap (red arrows) is seen within the lumen of the arch. The aortic root is partially visualized on the left side of the image.

Video of suprasternal notch view of aortic arch: The aortic arch is seen in the center-left of the image, and the ascending aorta is seen on the left-hand side of the image. A mobile dissection flap is seen in the lumen of the arch as a hyperechoic line which flutters with aortic pulsations.

Contrast-enhanced coronal CT scan of the aorta, confirms the aortic dissection beginning just distal to the aortic root (red arrows).

Photo demonstrates how to position the patient and orient the probe to perform the suprasternal notch view. The head is in extension, and the transducer is placed at the sternal notch pointing inferiorly.

## Discussion

Ascending AD is a rare, life-threatening diagnosis with varied presentations ranging from dramatic to subtle, which carries a mortality rate approaching 60% without surgical intervention.^1^,^2^ The mortality increases 1%–2% for every hour delay in surgical management.^3^ Several diagnostic imaging options exist: contrast-enhanced CT, magnetic resonance imaging (MRI), and transesophageal echocardiography (TEE), which all have excellent sensitivity and specificity for aortic dissection,^4^,^5^ but have important practical disadvantages in the emergency setting. MRI and TEE are time consuming, and may not be immediately available in some hospitals, especially outside business hours. CT and particularly MRI are not feasible for unstable patients, since both require the patient to leave the ED.

The reported sensitivity of standard transthoracic echocardiography for the detection of AD ranges between 59%–83%, but is higher for ascending AD.^4^,^7^ An emergency physician focused cardiac ultrasound protocol with particular assessment of dilation of the aortic root has been shown to improve diagnostic accuracy for AD and reduce time to intervention.^6^ A standard ED ETTE typically includes subxiphoid, parasternal, and/or apical 4-chamber windows. The SSNV is not routinely included with ETTE; however, it may improve both sensitivity and specificity for AD.^7^ As demonstrated in this case, the SSNV allowed the diagnosis of ascending AD to be made rapidly, permitting expedited communication with surgery and mobilization of the operating room resources.

With practice, the SSNV can be easily obtained in just a few minutes. It is ideally performed with the patient supine, though it may be performed with patients seated if they cannot lie flat. The patient’s neck must be in at least partial extension, which may be facilitated or augmented by placing a roll behind the patient’s shoulders, or by having patients hang their head over the top of the bed. This may be challenging or impossible for patients with restricted neck mobility, which is a limitation of the technique. The examination may also be more difficult in patients with hyperexpanded lungs, such as chronic obstructive pulmonary disease. The SSNV requires the use of an “echo” transducer (also known as a sector or phased-array transducer), due to its small contact face (or “footprint”). The suprasternal notch is fairly small and therefore provides a narrow window for imaging into which larger transducers cannot fit. The transducer is placed just above the suprasternal notch, pointed inferiorly into the thorax, as shown in the **photo**, and the imaging plane should be aligned with the long axis of the proximal aorta and aortic arch. This is accomplished by rotating the transducer slightly off of the frontal/coronal plane, roughly along an imaginary line connecting the sternal notch to the patient’s left scapula. The image will often need to be optimized by fanning the probe slightly anteriorly or posteriorly, and making small adjustments to the rotational axis to identify the best imaging plane.

Attention must be paid to the placement of the position indicator (also known as the “screen indicator”) on the ultrasound machine, and the location of the probe marker on the transducer. These must be correctly aligned such that the image is oriented to display the ascending aorta on the left side of the screen, the arch roughly in the center of the screen, and if the descending aorta is visualizable, it should be seen on the right side of the screen. The default location of the screen indicator varies depending on the exam settings (“presets”) selected by the user. “Cardiac” exam settings usually place the screen indicator by default in the upper-right side of the screen. In this case, for the image to be properly displayed, the probe marker should be oriented towards the patient’s left side. Conversely, if the screen indicator is located in the upper-left side of the screen, the probe marker should be oriented towards the patient’s right side. Additional instructional resources for performing the SSNV can be found in the references section.^8^,^9^

This case illustrates how addition of the suprasternal view to ETTE can assist with timely identification of an ascending aortic dissection. This may be particularly useful in patients with hemodynamic instability, contraindications to contrast, or when definitive diagnostic tests are not available or feasible.

## Supplementary Information



















## References

[1] Diercks DB Promes SB Schuur JD Clinical policy: critical issues in the evaluation and management of adult patients with suspected acute nontraumatic thoracic aortic dissection Ann Emerg Med 2015 65 1 32 42 10.1016/j.annemergmed.2014.11.002 25529153

[2] Hagan PG Nienaber CA Isselbacher EM The International Registry of Acute Aortic Dissection (IRAD): new insights into an old disease JAMA 2000 283 897 903 10.1001/jama.283.7.897 10685714

[3] Milewicz DM Stopping a killer; improving the diagnosis, treatment, and prevention of ascending aortic dissections Circulation 2011 124 18 1902 1904 10.1161/CIRCULATIONAHA.111.059337 22042924

[4] Meredith E Masani N Echocardiography in the assessment of acute aortic syndromes Eur J Echocardiogr 2009 10 1 i31 9 10.1093/ejechocard/jen251 19131497

[5] Rosenberg H Al-Rajhi K ED ultrasound diagnosis of a type B aortic dissection using the suprasternal view Am J Emerg Med 2012 30 9 2084e1 5 10.1016/j.ajem.2011.11.012 22244228

[6] Pare JR Liu R Moore CL Emergency physician focused cardiac ultrasound improves diagnosis of ascending aortic dissection Am J Emerg Med 2016 34 3 486 492 10.1016/j.ajem.2015.12.005 26782795

[7] Sparks SE Kurz M Franzen D Early identification of an atypical case of type A dissection by transthoracic echocardiography by the emergency physician Am J Emerg Med 2015 33 985e1 3 10.1016/j.ajem.2014.12.024 25704184

[8] Binder T Frimmel N Altersberger M 2.3.4 Suprasternal window 123sonography https://www.123sonography.com/ebook/suprasternal-window Accessed October 20, 2019

[9] Medmastery Echocardiography essentials: mastering the suprasternal view of the aorta YouTube https://www.youtube.com/watch?v=gv6yZNOIchE. Published April 18, 2017 Accessed October 20, 2019

