# Supplementary figures and images for: Ascending Thoracic Aortic Dissection: A Case Report of Rapid Detection Via Emergency Echocardiography with Suprasternal Notch Views
Source: J Educ Teach Emerg Med. 2020 Apr 15;5(2):V14–8. doi: 10.21980/J8WW6W (PMC10332572; doi:10.21980/J8WW6W)

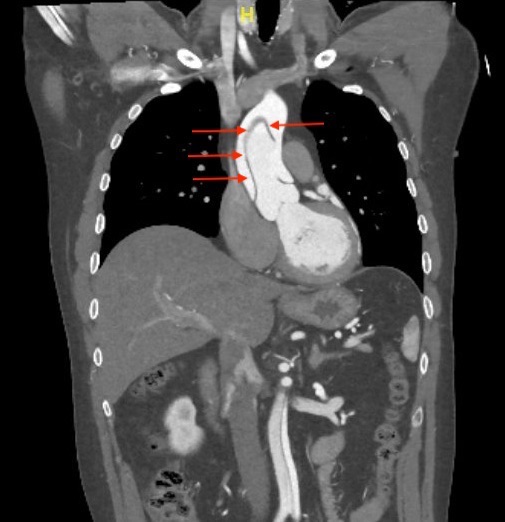

Supplement: Supplementary file 3 [file jetem-5-2-v14-supp3.jpg]

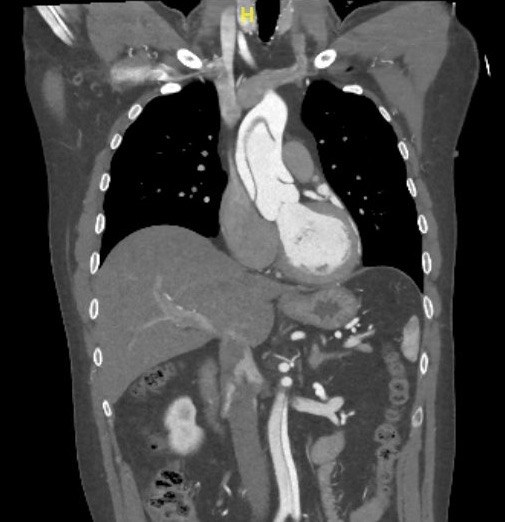

Supplement: Supplementary file 4 [file jetem-5-2-v14-supp4.jpg]

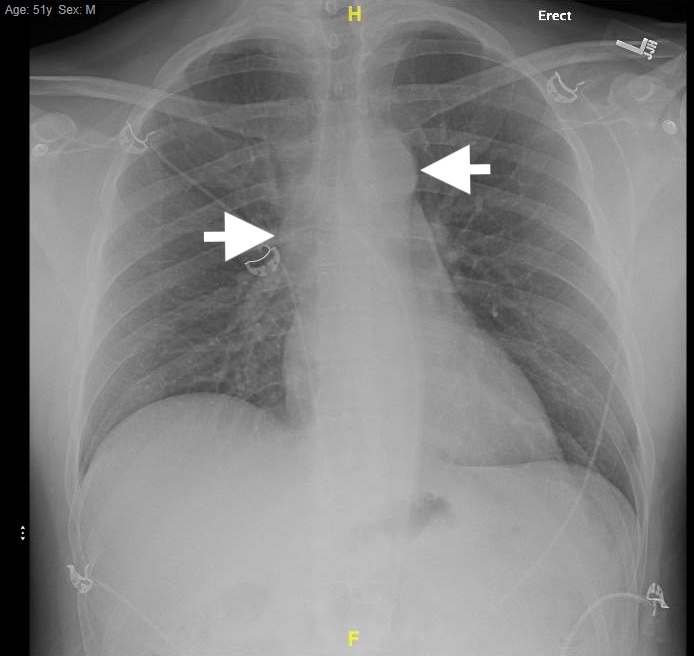

Supplement: Supplementary file 5 [file jetem-5-2-v14-supp5.jpg]

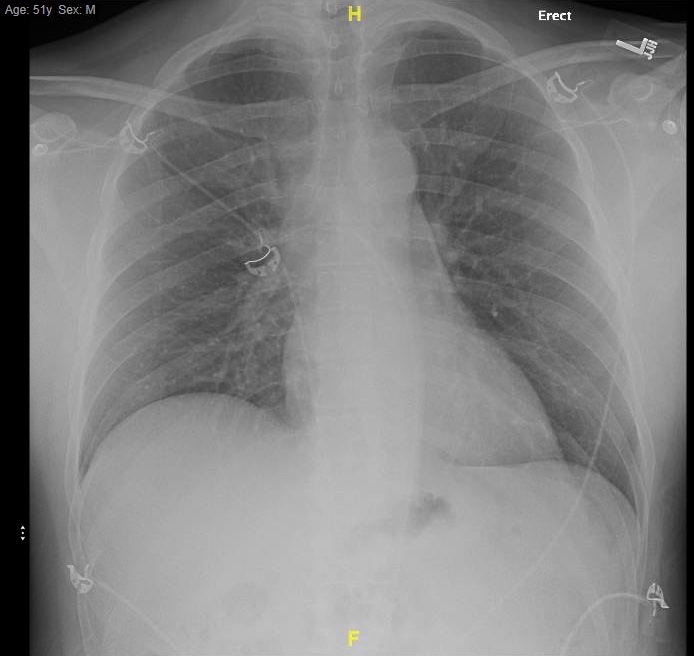

Supplement: Supplementary file 6 [file jetem-5-2-v14-supp6.jpg]

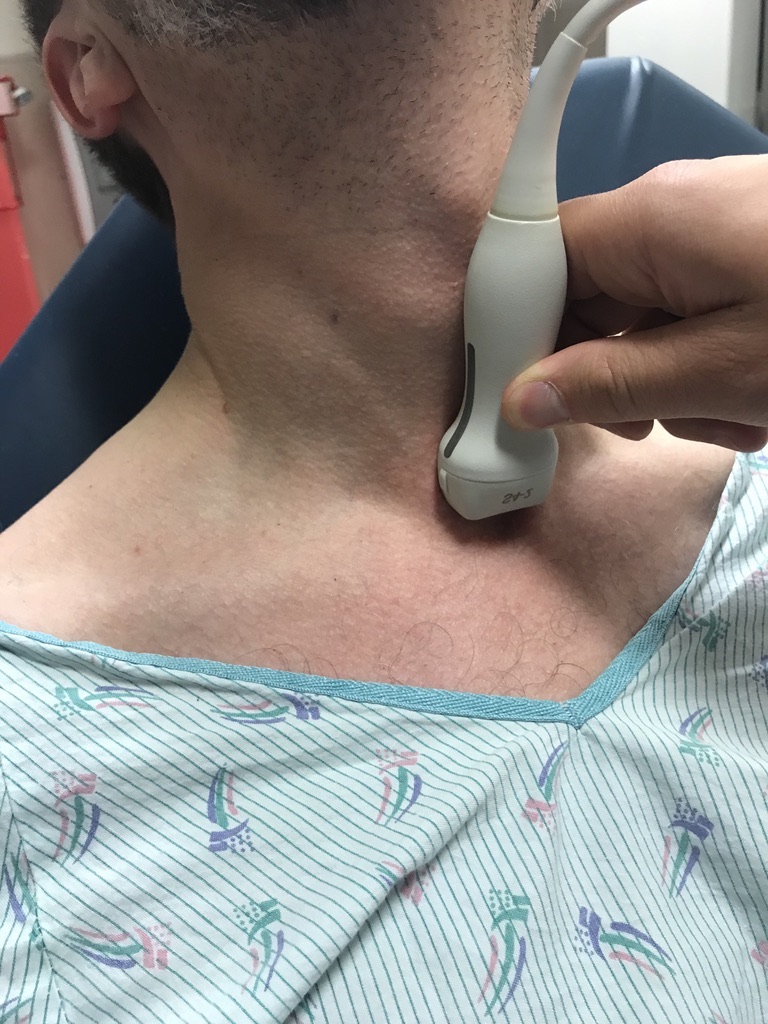

Supplement: Supplementary file 7 [file jetem-5-2-v14-supp7.jpg]

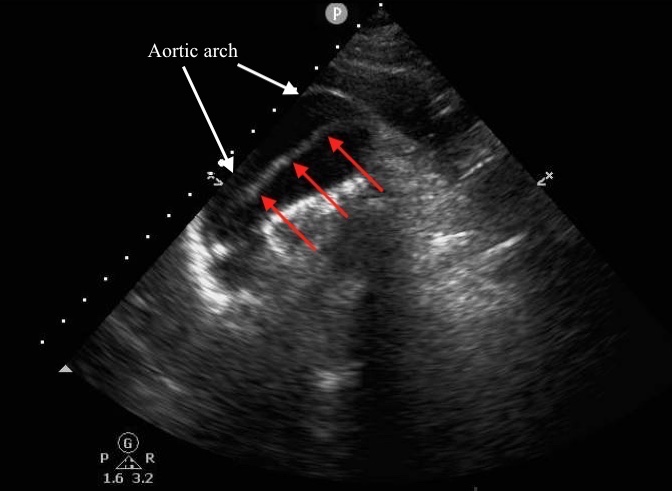

Supplement: Supplementary file 8 [file jetem-5-2-v14-supp8.jpg]

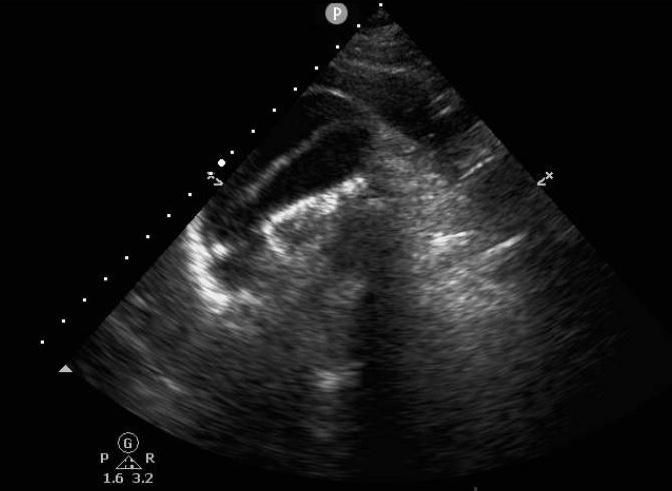

Supplement: Supplementary file 9 [file jetem-5-2-v14-supp9.jpg]
